# Racism and mental health within humanitarian and development organisations: perspectives from in-house mental health professionals

**DOI:** 10.3389/fpsyg.2026.1771077

**Published:** 2026-08-12

**Authors:** Hannah Strohmeier, Alice Gritti, Aza Stephen Allsop, Jasmina Schroff, Lukoye Atwoli

**Affiliations:** 1 Center for Global Health and Institute of International Health, Charité University Medicine Berlin, Berlin, Germany; 2 School of Health in Social Sciences, The University of Edinburgh, Edinburgh, United Kingdom; 3 Department of Psychiatry, Yale University, New Haven, CT, United States; 4 Center for Collective Healing, Department of Psychiatry and Behavioural Sciences, Howard University, Washington, DC, United States; 5 Zentrum für Internationale Friedenseinsätze, Berlin, Germany; 6 Department of Medicine, Medical College East Africa and Brain and Mind Institute, The Aga Khan University—Kenya, Nairobi, Kenya

**Keywords:** diversity, humanitarian, inclusion, mental health, racism, staff health, thematic analysis, workplace

## Abstract

**Introduction:**

Racism is recognised as a concern within humanitarian and development (HD) organisations, yet little research has examined its implications for staff mental health. Notably, the perspectives of in-house mental health professionals (MHPs)—a key but understudied group—have remained absent from the literature. This study addresses this gap by asking three questions: First, how do MHPs perceive and personally experience racism within HD organisations? Second, in what ways does racism affect the mental health of staff, and how do MHPs navigate their role in addressing these impacts? Third, how do MHPs evaluate existing organisational responses to racism, and what changes do they consider necessary to strengthen anti-racism efforts and link these to staff well-being?

**Methods:**

Semi-structured interviews were conducted with ten currently employed or recently retired MHPs across United Nations entities and an organisation belonging to the Red Cross/Red Crescent Movement. Participants were recruited via organisational circulation and snowball sampling. Data were analysed using inductive thematic analysis.

**Results:**

Three themes were identified. (1) *Experiences and impacts of racism among HD staff*: MHPs described racism as pervasive yet rarely discussed, manifesting in inequities between national and international staff, donor-related privilege, exclusion, and substantial mental health consequences. Staff often coped privately, seldom using formal in-house psychosocial support. (2) *Roles and professional boundaries of MHPs*: Racism frequently appeared as a latent factor behind other complaints. MHPs employed a range of strategies, such as validating clients’ experiences and outlining available response pathways. They recognised that aspects of their own identity may initially shape staff members’ help-seeking behaviour. (3) *Organisational responses and constraints*: MHPs acknowledged progress, yet anti-racism efforts were viewed as insufficient and disconnected from staff-care systems. Recommendations included a shift from reactive to preventive action and training.

**Discussion:**

Racism in HD organisations emerges as widespread yet insufficiently addressed, with its mental health impacts largely managed in private. Findings indicate the need for integrated approaches that link anti-racism and staff-wellbeing strategies, recognise racism as an occupational health issue, and include MHPs in organisational transformation efforts. This study provides evidence from a hard-to-reach and rarely examined group, and highlights critical directions for institutional change.

## 1 Background

Although distinct in their focus, humanitarian and development (HD) work is closely interconnected in practice. Many prominent organisations—including entities within the United Nations (UN) system, the International Red Cross and Red Crescent Movement (hereafter “the Movement”), and international non-governmental organisations (INGOs)—frequently operate with multiple mandates, engaging in both humanitarian response and development programming (and in some cases also peace-related efforts) (Fanning and Fullwood-Thomas, 2019). The HD workforce is extensive, comprising over 630,000 individuals engaged in humanitarian crisis settings alone in 2020, the latest year for which data are available (ALNAP, 2022). It is also notably diverse, encompassing staff of different nationalities, ethnicities, genders, religions, and other identity-shaping characteristics. This diversity is a defining feature of the sector that, if managed well, can be beneficial to both workers and organisations (Tamunomiebi and John-Eke, 2020). At the same time, it underscores the importance of preventing and addressing issues of equity and inclusion within the workplace. Against this backdrop, global racial justice movements—particularly those catalysed by the 2020 killings of George Floyd, Breonna Taylor, and others in the United States—resonated within HD organisations and contributed to heightened scrutiny of inequality and discrimination, including within their own ranks (UN holds dialogues, 2020; Rejali, 2020). These debates have gained traction in both scholarly and policy spaces, prompting calls for both anti-racist transformation of organisations and the decolonization of aid more broadly (e.g., Open Letter to Senior, 2020; Ali and Murphy, 2020; Currion, 2020; Elahee, 2021; Bian, 2022; Slim, 2022; The International Development Committee, 2022; Khan et al., 2023; Strohmeier et al., 2024a; Strohmeier et al., 2024b; Dilger et al., 2025). Yet despite increasing calls for change from scholars and aid workers themselves, empirical research remains limited, rendering the broad field of racism within HD organisations an under-explored topic in academic literature (Strohmeier et al., 2024b).

### 1.1 Conceptualisation of racism

The conceptual framing presented here draws in part on related work by Strohmeier and Karunakara (In press), adapted for the present paper.

Different definitions of racism exist, and there are longstanding and ongoing debates in the literature, including across different disciplines (e.g., Sawrikar and Kat, 2010; Worther, 2020; Urquidez, 2021). Importantly, however, there is consensus that race is not biologically grounded but rather a social, political, and historical construct that emerged in the context of European expansion and colonialism (e.g., Omi, 2001; Kluge et al., 2020; Morey, 2023; Schnakenbourg, 2023).

The American Psychological Association (2023) understands racism as a system through which opportunities and social value are distributed according to racialised physical characteristics, influencing both interpersonal interactions and structural access to resources, and producing persistent disadvantages for marginalised racial groups. It also describes racism “a form of prejudice that generally includes negative emotional reactions to members of the group, acceptance of negative stereotypes, and racial discrimination against individuals; in some cases it leads to violence” (APA, 2023). The UN writes that “racism includes attitudes, practices and beliefs rooted in ideas or theories of superiority, as a complex of factors, which produce discrimination and exclusion” (Task Force on Addressing Racism and Promoting Dignity for All in the United Nations Secretariat, 2021, p. 7). This definition further emphasizes that racism manifests in a range of forms, including stereotyping, harassment, negative remarks, and hate crimes. It may also be embedded in cultural norms, educational systems, values, and beliefs, shaping workplace cultures and behaviours.

Building on these definitions, racism operates across multiple, interconnected levels and can take both overt and subtle forms. These include internalised racism (where individuals adopt and reproduce racist beliefs), interpersonal racism (between individuals), institutional racism (embedded in organisational policies and practices), and structural racism (referring to broader social, economic, and political systems that sustain racial inequalities) (Rodriguez-Knutsen, 2023). In this context, it is important to note that racist attitudes may differ by place, and that the expression of institutional racism is likely to vary across sectors and institutional settings, underscoring the need for context-specific analysis (Trennery et al., 2024).

### 1.2 Racism within HD organisations

Currently, most of the available, systematically collected data on racism originates from internal assessments conducted by HD organisations themselves. One key example is the secondary analysis of racial discrimination data within UN offices in Geneva (Strohmeier et al., 2024b), which was originally gathered by the UN staff union following the events of 2020. This analysis, based on responses from 1,251 staff, consultants, and interns, found that approximately one-third of the participants had personally experienced racial discrimination in the workplace (34.4%). Survey participants were also asked how racial discrimination had manifested, and the most frequently reported answer was a negative impact on career advancement (66.2%), followed by exclusion from work events (37.9%). A significant proportion reported experiencing verbal abuse (26.8%) and/or being falsely accused or criticised for wrongdoing (25.7%). Regarding actions taken in response to racial discrimination, over half (57.4%) reported taking no action. The most frequently cited reasons for inaction were a lack of trust in the organisation’s recourse mechanisms (67.3%) and fear of retaliation by those involved (55.5%). Among those who did take action, many spoke with colleagues (42.1%), and some reported the incident to a supervisor (16.4%). Only a few submitted an official complaint (1.8%).

The UN Secretariat in New York launched a similar survey in late 2020, aiming “to assess staff perceptions on the extent of racism and racial discrimination in the Organisation” (Task Force on Addressing Racism and Promoting Dignity for All in the United Nations Secretariat, 2021). The publicly available summary of the survey, which drew responses from over 8,000 Secretariat staff (representing 22% of total staff), revealed similarly stark findings: one in three respondents reported having experienced discrimination. Of those, nearly half (49%) stated that these experiences occurred occasionally, while more than one in five (21%) reported experiencing discrimination frequently.

Beyond these institutional surveys, publicly available, systematically collected qualitative and quantitative data on racism within the HD sector remain limited. Aid Works, a small social enterprise, is one of the few actors that have attempted to collect data by conducting a rapid survey in 2020. The survey targeted aid workers of all nationalities and ethnicities, whether based in head offices or in aid-recipient settings, and regardless of whether they had experienced racism at work. Responses were gathered from 286 participants across 63 countries, the majority of whom were employed by international NGOs. While the sample size was small and the methodology subject to several limitations, the findings nonetheless offer some insights. Of the respondents, 143 reported experiencing racism in the workplace within the previous year, with more than two-thirds indicating they had faced three or more incidents during that time. The most frequently reported forms of racism included microaggressions (81%), pay or benefit discrimination (62%), being overlooked for training or promotion (54%), and being discouraged from expressing opinions (54%). Over half of those who experienced racism did not report the incidents (53%), primarily due to perceptions that the issues would not be addressed effectively (70%) and fears of negative professional consequences (58%) (Ali, 2021).

There are at least three compelling reasons—legal, moral, and economic—for organisations to actively address racism within their ranks (Strohmeier et al., 2024b). First, racial discrimination is prohibited under international law (UN General Assembly, 1965). Second, organisations have a duty of care to protect staff from harm (Guttry et al., 2018). Third, compromised wellbeing of staff can directly undermine organisational functioning—for instance, through increased sick leave and staff turnover—and, in turn, may also damage institutional credibility with donors and communities (Prom-Jackson, 2023; Strohmeier et al., 2024b). And yet, all of the above cited studies, alongside other reports and literature related to this topic (e.g., McVeigh, 2020; Bian, 2022; Slim, 2022; The International Development Committee, 2022; Prom-Jackson, 2023), point to a consistent trend: racism appears to remain both widespread within HD organisations and significantly underreported. Furthermore, its consequences—particularly its effects on staff mental health—are rarely captured in existing data collections.

### 1.3 Mental health impacts and policy silos

A substantial body of evidence—predominantly derived from studies conducted in Western contexts—demonstrates a clear association between racism and poor health, with particularly strong links to adverse mental health outcomes. These include affective disorders (particularly depression), anxiety disorders, substance use disorders, and psychotic symptoms in some populations (e.g., Paradies et al., 2015; Schouler-Ocak et al., 2021; Schouler-Ocak and Moran, 2022; Webb et al., 2022). Increasingly, the literature also conceptualises racism as a traumatic stressor, with some individuals experiencing racial discrimination as psychological trauma (Polanco-Roman et al., 2016; Bird et al., 2021; Cénat, 2023). Studies examining racism in the workplace have similarly identified adverse impacts on employee mental health (Triana et al., 2015). As a result, racism is recognized in the health community “as one of the most consequential transnational phenomena to impact the health and lives of afflicted communities globally” (Erondu et al., 2023, p. 1834). These negative (mental) health effects of racism—including experiences of perceived discrimination (Pascoe and Smart Richman, 2009)—arise from multiple, intersecting pathways. First, racism functions as a psychosocial stressor: discrimination and exclusion can trigger sustained stress responses, which are known to adversely impact both physical and mental health. Chronic stress has been linked to dysregulation of the hypothalamic–pituitary–adrenal (HPA) axis, immune system impairment, and increased risk for a range of psychological conditions (Berger and Sarnyai, 2015). In addition, racism can influence health through behavioural pathways. It is associated with increased engagement in health-risk behaviours (e.g., substance use) and reduced participation in health-promoting behaviours (e.g., exercise). Racism may also contribute to the underutilization and delayed access of health services, resulting in late diagnoses and treatment. Additionally, health care professionals may hold unconscious biases or prejudices, which can further compromise the quality of care—ultimately leading to poorer health outcomes over time (Pascoe and Smart Richman, 2009; Schouler-Ocak et al., 2021). These effects may be further compounded by intersectionality, which can shape both the intensity of exposure to racism and the resources available to mitigate its health impacts (Crenshaw, 1991).

Despite the growing body of evidence linking racism and mental health, institutional policies within HD organisations tend to address these issues in parallel rather than as interrelated concerns. For instance, the UN Strategic Action Plan on Addressing Racism (2021) includes among its measures the provision of “education and training opportunities for staff counsellors and other support personnel (e.g., peer support).” Additionally, one of the anticipated outcomes of the institutionalization of the “Racial Justice Focal Points Volunteer Network” is improved access to support services, including those related to mental health. While these provisions are notable, the plan itself does not explicitly address the psychosocial or mental health impacts of racism on staff. Similarly, although the UN System Mental Health Strategy for 2024 and beyond (United Nations, 2024) briefly refers to some risk factors for poor mental health in its annex (e.g., bullying, harassment, and abuse), it remains largely unspecific and does not explicitly mention racism. It is unclear whether HD organisations meaningfully connect racism and mental health in practice.

### 1.4 The role of in-house mental health professionals

Large HD organisations—including entities within the UN system, the Movement, and prominent INGOs—employ professionals tasked with safeguarding the mental health and psychosocial well-being of staff. Depending on the organisation, these roles are designated by various titles, such as “stress counsellor,” “staff counsellor,” “staff health officer,” or “staff mental health and psychosocial support delegate”. For reasons of clarity and confidentiality, this paper refers to them collectively as mental health professionals (MHPs). Typically, MHPs are licensed psychologists or medical doctors, and their responsibilities span a wide range of functions, including psycho-education and awareness-raising, critical incident management, and the provision of psychosocial support. While they generally do not offer long-term psychotherapy, MHPs may refer staff to external services and support formal procedures such as sick leave when required (e.g., Mason, 2022; Well-Being and Mental, 2026).

As providers of staff care and embedded members of institutional structures, MHPs occupy a unique role within HD organisations. This positioning makes them a valuable source of insight into how racism manifests in the workplace and how it intersects with mental health. However, their perspectives remain largely absent from the academic literature. To date, little is known about how MHPs perceive and navigate racism—whether in their professional work with staff, within the wider organisational culture, and through their personal experiences. Likewise, it remains unclear to what extent they feel knowledgeable and equipped to address racism as a mental health concern, or how their ability to do so may be shaped by help-seeking behaviour of staff, which may be influenced by stigma, distrust, or fear of repercussions; professional boundaries; or institutional politics and organisational culture.

Despite their relevance, MHPs represent a “hard-to-reach” population for researchers. Their numbers are relatively small across the sector, they often bear responsibility for large staff populations, and they operate under high workloads and strict confidentiality standards. These factors contribute to time poverty and professional discretion that may deter participation in research. In turn, the scholarly literature on humanitarian staff health has largely overlooked this professional group—studies focusing specifically on MHPs remain, to our knowledge, virtually nonexistent.

### 1.5 Study objective and research questions

This study aimed at addressing gaps in the literature on racism and mental health within HD organisations by exploring the perspectives of in-house MHPs. It asked three central research questions: First, how do MHPs perceive and personally experience racism within HD organisations? Second, in what ways does racism affect the mental health of staff, and how do MHPs navigate their role in addressing these impacts? And third, how do MHPs evaluate existing organisational responses to racism, and what changes do they consider necessary to strengthen anti-racism efforts and link these to staff well-being? In doing so, this study applies a conceptually broad lens that encompasses lived experiences, psychological and emotional impacts, and institutional critique. This includes participants’ own interpretations of racism, even where these overlap with or are difficult to distinguish from related forms of discrimination.

## 2 Methods

### 2.1 Eligibility and recruitment strategy/sampling

The data presented in this article were collected as part of a broader research project on racism and mental health conducted at Charité—Universitätsmedizin Berlin between 2022 and 2024. Eligible participants for this study component were MHPs currently or previously working for an HD organisation and holding roles focused on staff care. To recruit participants, the first author contacted the UN Headquarters in New York and one organisation within the Movement, requesting that they circulate the invitation for study participation among eligible staff. The organisation within the Movement supported the study and, in June 2023, sent an email invitation to their MHPs, followed by a reminder email 2 weeks later. The UN did not respond to the request for support. The first author then utilized their professional networks within the UN and approached eligible staff through snowball sampling. In terms of sample size, this study is situated within Braun and Clarke’s (2020) critical perspective on the use of saturation in qualitative research. Accordingly, it moves away from a positivist standpoint and instead adopts an approach, which emphasises “information power” (Sim et al., 2018) rather than data saturation: “the more relevant information a sample holds, the fewer participants are needed” (Braun and Clarke, 2019, p. 210).

### 2.2 Data collection and analysis

Between June 2023 and May 2024, the first author conducted semi-structured online and in-person interviews with 10 MHPs on the topic of racism and mental health in the workplace. The interview guide was developed based on existing research on workplace racism and specific research questions of interest to the study. It was reviewed by a diverse team of scholars and humanitarian practitioners and pilot-tested prior to data collection. To build rapport, the first author shared information about their research project, as well as consulting experience with the UN. The open-ended interview questions addressed a wide range of topics, including personal and organisational understanding of racism, the effects of racism in the workplace, and strategies to prevent and address racism and its adverse consequences. Specifically, they asked, for example: “How does racism manifest within your organisation—do you see any patterns?,” “Please provide an estimate: how many of the people who seek your support do so because of the experience of racism?,” “Based on your expertise as MHP, how do you think the experience of racism affects staff’s mental health and well-being?,” and “In what way do you think your own identity influences who seeks your support?”.

The interviews lasted between 40 and 80 mins, with most being close to one hour. They were recorded and transcribed. The first author then conducted a thematic analysis with the aid of NVIVO software, following the six-step approach outlined by Braun and Clarke (2021). In line with a reflexive approach to thematic analysis, coding was treated as an interpretative and iterative process shaped by the researcher’s engagement with the data and the subjective engagement and interpretative lens they bring to the procedure, rather than as a process requiring coding agreement across multiple analysts (Braun and Clarke, 2019, 2020). An inductive approach to coding was applied, with a focus on the semantic/manifest (as opposed to latent) meanings in the data (Braun and Clarke, 2012). To maintain a more focused analysis, text was assigned to one code only (‘exclusive coding’) (Joffe and Yardley, 2004). Recognising that qualitative data often contain overlapping meanings, the first author considered alternative interpretations and reflected on the coding decisions continuously throughout the analysis, revisiting and refining codes where appropriate to ensure that more nuanced meanings were not overlooked.

### 2.3 Study participants

Seven of the 10 MHPs identified as men, most were between 30 and 49 years old, and held MA degrees (or equivalent) in the fields of medicine and psychology. Half of the sample worked for the UN, and the other half for an organisation within the Movement. Participants held nationalities from countries in the regions of Africa, Asia and the Pacific (AP), Latin America and the Caribbean (LAC), and Western Europe and other States (WEO). At the time of the interview, most were serving in duty stations located in AP and WEO (see Table 1).

**Table 1:** Sample characteristics of the mental health professionals interviewed.

**Gender**	Men	7
	Women	3
**Age**	30–39	4
	40–49	2
	50–59	1
	60+	3
**Education**	MA	7
	PhD	3
**Region of origin**	Africa	2
	Asia and the pacific	3
	Latin America and the Caribbean	2
	Western European and other States	3
**Organisation**	International red cross and red crescent movement	5
	United Nations	5
**Latest duty station**	Africa	1
	Asia and the pacific	3
	Western European and other States	3
	Other*	3

*Two interviewees had recently retired, and one was in between jobs at the time of the interview. Source: authors.

### 2.4 Confidentiality and ethical approval

As noted earlier, MHPs constitute a comparatively small professional group within the HD sector. To protect participant confidentiality, we removed names and organisational affiliations, and replaced countries of origin with broader regional descriptors. We also refrained from presenting combinations of demographic characteristics. Participants were assigned numbers, and personal background details were not included in interview quotes. While identity markers are important in the context of racism-related research, this decision was made to safeguard anonymity and avoid deductive disclosure (Ethical considerations associated, 2022). Ethical approval for the study was granted by the Charité Ethics Committee in April 2023 (EA1/081/23). All participants provided informed consent prior to the interview.

### 2.5 Researcher positionality

The first author, who identifies as a white German woman, conducted the interviews and carried out the data analysis and manuscript drafting. During data collection, no explicit indications were observed that her positionality shaped participants’ accounts or interactions across participants, irrespective of their backgrounds. At the same time, the authors acknowledge that such influences may be subtle or unarticulated and cannot be fully ruled out, and that this assessment is itself situated. Co-authors contributed input and feedback informed by their diverse social identities and professional perspectives, which helped to critically interrogate assumptions and strengthen the interpretation and discussion of the findings.

## 3 Results

The analysis identified three main themes: “Experiences and impacts of racism among HD staff,” “Roles and professional boundaries of in-house MHPs,” and “Organisational responses and constraints.” Each of the themes included sub-themes, as shown in Table 2.

**Table 2:** Themes and sub-themes.

**Themes**	1 Experiences and impacts of racism among humanitarian and development staff	2 Roles and professional boundaries of in-house mental health professionals	3 Organisational responses and constraints
**Sub-themes**	1.1 Manifestations of racism	2.1 Helping with racism and mental health problems	3.1 Evaluation of organisational responses to racism and mental health
	1.2 Mental health impacts of racism	2.2 Knowledge and preparedness to address racism	3.2 What organisations should do
	1.3 Individual coping strategies	2.3 MHPs identities’ impact on help-seeking	
	1.4 Seeking formal in-house psychosocial support	2.4 MHPs’ personal experiences of racism	

Source: authors.

### 3.1 Theme 1: Experiences and impacts of racism among humanitarian and development staff

#### 3.1.1 Sub-theme 1.1: manifestations of racism

MHPs identified racism as a persistent issue within organisations, taking systemic, institutional, and interpersonal forms. These were often tied to bodily markers such as skin colour—the term participants themselves used—alongside nationality, ethnicity, and internal group dynamics. A recurring concern was the sector-wide divide between national and international staff. National staff, despite often being highly qualified, were reported as structurally disadvantaged—receiving lower pay, fewer rights, and limited opportunities for career advancement compared to international staff. As one participant put it, national staff “never move in their degree” (Participant 1). The same participant described how these inequities are sometimes reinforced by discriminatory attitudes, prejudice, and stereotypes. Reflecting critically on these dynamics, they reported that some international European staff expressed the belief that “national staff from the developing world” do not deserve international positions, stating: “because the education is less, so their capacity is less.” They also noted that national staff are at times labeled with harmful stereotypes such as “thieves,” “bad,” and “lazy people who do not like working” (Participant 1). According to this participant, such perceptions are further fueled by a lack of understanding of the local culture and country context. As they explained:

*‘Someone in Afghanistan, a French [staff member] in Afghanistan, he has never actually seen Afghanistan. He saw it from the airplane. Or Baghdad for example: they have never really been in Baghdad or interacted with the people. (…) And the scene in Africa, in Uganda and Ethiopia, I hear the same issue’* (Participant 1).

These dynamics also create conditions in which racialized staff feel compelled to over-perform and continually demonstrate their competence:

*‘You cannot be normal, or you cannot have a bad day, or you cannot miss one deadline. Because of your colour, because of your origin, it will be exacerbated (…). You always have to be better, you always have to have a better analysis of the context’ *(Participant 5).

Another double standard was reported in institutional responsiveness to crises, where national staff or staff from less influential countries received less protection and support in incidents such as hostage situations. As one MHP explained, for staff from countries like Yemen, Bangladesh, or Egypt, “the UN would not care,” whereas staff from “strong countries politically, the main donors of the UN” were more likely to receive action and support from their organisation (Participant 1).

The role of nationality further complicated how racism was experienced. While not always explicitly racialized, one MHP noted that some nationalities received preferential treatment due to donor influence—donors who “want to provide some opportunities to their citizens, to firstly introduce [them] to the UN system” (Participant 6).

Similarly, staff were reported to gravitate toward those from their own country and cultural background, especially among international staff based abroad, shaping both professional networks and informal support. As one MHP explained:

*‘The problem in the UN is not (…) white is always racist, and Black [people] are always victims (…). It’s the sub-group dynamics, (…) those are the main power dynamics. The preference is usually based on similar groups — if you are Indian, then you will go towards the Indian (…) because it’s more (…) safe’* (Participant 1).

At the same time, intra-national discrimination also surfaced. Another MHP noted that conversations within human resources about national staff recruitment were influenced by security concerns, particularly when certain groups—such as clans—were under or over-represented. This reflects a different way in which local political and social dynamics intersect with institutional practices.

On a more interpersonal level, racism manifested through subtle forms of social exclusion and division. One participant described a commonly observed situation:

*‘One example is when people do not sit to eat with you, for instance, because you are (…) from a different colour. They cannot invite you out because of that’* (Participant 2).

#### 3.1.2 Sub-theme 1.2: mental health impacts of racism

While one MHP reflected uncertainty—“I do not know if racism has an impact on mental health” (Participant 8)—the majority clearly affirmed the link. Indeed, drawing on both professional observations and broader reflections, MHPs described racism as having significant and often severe effects on mental health.

A frequently mentioned impact was the experience of exclusion, isolation, and lack of validation, leaving staff emotionally drained. In addition to psychological strain, MHPs observed and anticipated a range of symptoms, disorders, and syndromes—including sadness, feeling threatened, anxiety, depression, burnout, and, in more acute cases, post-traumatic stress disorder (PTSD) and even psychotic episodes. One MHP described the cumulative toll in their practice: “It’s devastating (…). I have seen people with PTSD, people with anxiety, (…) people start to take medications just to be able to cope” (Participant 3).

Another MHP emphasized how persistent racial abuse from someone higher in the organisational hierarchy could escalate into serious mental health breakdowns: “People might develop anything—depression and anxiety (…) because of this racist manager, people might develop (…) a psychotic episode or whatever it is, absolutely” (Participant 7).

Beyond clinical diagnoses, MHPs highlighted how racism affects broader personal and professional functioning. Participants described staff becoming paralysed in their social lives, experiencing tension in family or romantic relationships, and losing motivation at work, which, in turn, can lead to underperformance or withdrawal from responsibilities.

What emerged as particularly harmful was the chronic, day-to-day nature of exposure to racism. Rather than isolated incidents, MHPs emphasized that ongoing power asymmetries, especially when linked to a direct manager or structural exclusion, posed a continuous threat to staff well-being. As one MHP put it:

*‘I just want to emphasise that racism is very diffused, it’s a silent process that starts as you step into the gate and then, until you leave and come back home, it’s constantly there. But nobody speaks about it (…). Sometimes it’s harmful. Sometimes it’s less harmful’* (Participant 3).

#### 3.1.3 Sub-theme 1.3: individual coping strategies

Coping with racism was described by MHPs as individualised, shaped by culture, personal agency, and the silence that often surrounds racism in the workplace. A recurring issue was that racism is rarely spoken about openly within organisations.

Cultural background was mentioned as a key factor influencing how individuals process and respond to racism. In some cultural contexts, a tendency to internalize such experiences was observed, rather than speaking out. One MHP, referring to “the Asian culture,” noted: “So people will definitely go to themselves. They will not speak out about it” (Participant 7). This was contrasted with their own cultural background: “Where I’m from (…), people are very talkative, they would like to bring it, and will make conflict.”

Furthermore, faith, family, and tradition were seen as important sources of resilience. As one MHP noted, in some duty stations, staff leaned heavily on religion and familial support: “The resilience of these people is mostly based in family and religion. And it works for them most of the time” (Participant 10).

Other coping strategies included avoidance, such as limiting time spent with the source of distress. One MHP described a colleague who managed their exposure to a manager by shifting their lunch hour: “I have a colleague who (…) takes the lunch break at a different time, just because that means one hour for the manager, one hour for them. That’s two out of eight hours (…) without that person together” (Participant 7).

Some staff, according to another MHP, coped by engaging in meaning-making activities or creating spaces of connection. They described how staff find alternative forms of fulfilment and belonging at work: “Some of them find the meaning on other things like book clubs with important discussions, or being a volunteer as a coach, or as ombudsperson. And finding a sense of community at the workplace, like creating the sense of community” (Participant 5).

Filing official complaints or reporting incidents was not among the typical coping mechanisms MHPs observed. As one put it: “They are very (…) intelligent, well informed people. They know what to do. They know their rights. They know how to go to the ethics office and open a fire whatever, but they choose not. Because, although formally you have things in place, it’s not how things work” (Participant 5). Instead of using formal mechanisms, staff sought support from the counsellor, attempted to move on, or ultimately chose to resign altogether: “They come to me, they cry, they talk about it, they might feel a little bit better. And then they just try to move on. And some of them lately have just left the organisation” (Participant 5).

#### 3.1.4 Sub-theme 1.4: seeking formal in-house psychosocial support

MHPs reported that staff typically approach them with general concerns—such as dissatisfaction with work, conflict with co-workers, stress related to supervision—but also trauma, loss of loved ones, and other personal struggles. Very few staff seek support explicitly for racism-related issues. Among those who gave estimates, one stated they have had no case at all, another reported only one case so far, and others estimated two to three cases in the last 3 years, “5–10% of the cases,” or “less than 10%.”

Multiple reasons were identified for the limited use of in-house psychosocial support in relation to racism. A central one was that racism is rarely spoken about within organisations. When MHPs themselves raise the topic—for example, in training sessions—staff may acknowledge it, but seldom bring it up voluntarily. This reluctance was further linked to both organisational culture and broader norms in the HD sector. Specifically, one MHP pointed to a culture of emotional suppression among aid workers: “We are ‘the guys’ and you should not break down (…). We must serve, and we control things” (Participant 7). They added, however, that this heroic self-image is unrealistic: “We know from the reality that it has never been like this” (Participant 7). The fast-paced, crisis-driven nature of humanitarian work was also said to leave little time or space for discussing racism. According to this MHP, staff often perceive there is no room to raise such topics when the focus is always on aid delivery and operational urgency.

Another major factor was a lack of safety. Staff were said to fear speaking openly about racism due to concerns about job security and contract renewal, and often lacked trust in human resources (HR) processes. In contrast, the MHP—as a medically protected and (more) neutral actor—was viewed as a safer point of contact. One MHP explained that staff might come to them rather than to HR to ask “how to make a complaint, where are the available resources?” (Participant 7), preferring the discretion of a clinical space.

A further explanation was the normalization of racism in humanitarian settings. As one MHP put it: “For them, it’s just normal (…). I am here, the environment is [like] that, and I will suffer this (…). So, they do not always come to complain to me” (Participant 5).

Only one MHP explicitly suggested that low case numbers might reflect a positive outcome of increased awareness: “Because I believe maybe the trainings are being effective” (Participant 9). Finally, one MHP cited findings from an internal survey suggesting that about half of the staff would generally prefer to seek external psychosocial support, further explaining the low uptake of internal counselling for racism-related distress and other mental health problems.

In addition, MHPs observed that clients do not always openly express what is going on—even in the protected setting of a counselling session. At times, they also may not fully recognise what is truly affecting them. As one MHP explained: “They come, complain about the work, (…) but it’s not the work. At the bottom line, it is being discriminated [against]” (Participant 3).

### 3.2 Theme 2: roles and professional boundaries of in-house mental health professionals

#### 3.2.1 Sub-theme 2.1: helping with racism and mental health problems

Several MHPs described how racism often appears as a latent factor, surfacing only through deeper exploration of psychological or somatic complaints. One MHP illustrated this by describing how racism can emerge beneath reports of workplace stress or physical symptoms such as headaches. To uncover these deeper dynamics, they emphasised the importance of asking broad, contextual questions: “Is there anything going on in the workplace? How’s your family doing? What are your financial responsibilities? Do you have any open loans?” Through such conversations, latent causes of distress sometimes become apparent: ‘Every now and then it was related to the hierarchy. So not always pure racism, but sometimes it was racism’ (Participant 4).

In response to these situations, MHPs described a variety of strategies for supporting clients. A common approach was to respect client agency while gently encouraging them to explore underlying issues. One MHP explained how they offer clients different ways of interpreting their situation: “I tell them, ‘based on what you shared with me, I think these are the different explanatory models (…). Which avenues do you want to travel?’” (Participant 4). Another MHP similarly emphasised supporting clients’ decision-making without pressuring action: “If it’s someone that is suffering a lot in a given team or with a given manager, then we try to explore possibilities of what they can do to protect themselves, if they want to do something about it or not” (Participant 5). Depending on the client’s needs and readiness, MHPs might also suggest organisational resources—such as referrals to the ethics office or the ombudsman—as options for formal follow-up.

Validation and reflection were also key aspects of MHP practice. One participant described how some staff only begin to recognise patterns after exploring past experiences: “Some of them actually will discover that they (…) have a history of similar incidents (…) and we start to acknowledge exactly what happened and how they should deal with it, or how they should stand for themselves” (Participant 6). Similarly, another participant highlighted the role of resilience when individuals’ experiences are validated: “Sometimes people come to me for only one session. Once I validate emotions, and that’s it, they continue their lives without my support. And without medication and without seeing the doctor or psychiatrist, for instance” (Participant 2). In their practice as described, MHPs help staff to make sense of cumulative experiences and consider how they might respond assertively or protect themselves.

However, some MHPs highlighted the importance of not rushing to label clients’ experiences as racism. Instead, they described a cautious and exploratory approach, focusing on the client’s emotional experience and psychological readiness first:

*‘Sometimes I put words, but I never put the word ‘racism’ (…), I use the word ‘discriminated against’. So when I’m talking about the feelings, (…) I’m saying, ‘Do you (…) maybe feel like this, because you are perceiving it as you being discriminated against (…)?’ And this is how we discuss it’* (Participant 2).

One MHP described an approach to their practice that emphasizes the individual’s response—rather than the event itself—as the key factor shaping emotional outcomes. As they explained: There is “nothing good” and “nothing bad” as such, but it is “the reaction to what happens to you that matters a lot” (Participant 9). They noted that while racism may be present, cognitive reframing techniques can help build resilience and even lead to what they viewed as positive outcomes: “Some people, even if it’s there, (…) may choose to look at [it] in a more positive light and not really bother about it. We take it differently (…). Some people may see that as a motivation to work harder, or to strive in life much much harder” (Participant 9).

Finally, one MHP made it very clear that while they provide support to individuals, they maintain medical discretion and professional boundaries, emphasizing that their role does not extend to driving organisational change: “I am in charge of the mental health of the colleague (…). I will support [them] on an individual level for self-care. But I’m not myself engaged in a battle or in investigation, (Participant 8).

#### 3.2.2 Sub-theme 2.2: knowledge and preparedness to address racism

None of the MHPs interviewed considered themselves experts on racism. Most described their knowledge as little to medium-level, often gained through general exposure to the topic, for example through the media. Some had encountered relevant content during university studies that covered discrimination, including racism, while others referred to organisational onboarding courses, including modules on ethics and intercultural work environments. However, as one MHP pointed out, this is “not the same as addressing racism” (Participant 4).

When it came to workplace resources, several MHPs noted a gap: while in some organisations, tools exist for handling cases of sexual violence or harassment, racism-specific guidance or training was lacking across the board. Supervision and intervision were available in some organisations, and while not focused on racism, MHPs said these spaces allowed for regular discussion of complex cases with colleagues, including those involving discrimination.

Importantly, several MHPs said they were attuned to the issue not because of training but because of personal lived experience. As one shared: “I’m very much aware of it. I notice [it] happening all the time, sometimes no expression, just kind of nonverbal. You know, the way people look at you, the way they greet you” (Participant 3). In the absence of formal instruction, many described drawing on their personal and professional backgrounds to navigate situations involving racism. As one put it:

*‘Well, yeah, this is complicated, because at the end, you rely on your own background and your own capacity to deal with another human being. So you try to make things equal and empathise. And maybe offer to talk in small groups, or to do by yourself some kind of session to talk about these issues with people that may be involved. But this [is] something really kind of individual, from your own initiative’* (Participant 10).

Notably, this study prompted reflection among some participants. For example, one MHP described the interview itself as an “eye opener” that raised new questions and awareness (Participant 2). Another commented, “I think there is a need to have this” (Participant 7), referring to training or resources focused on racism in the workplace, and how to support clients.

#### 3.2.3 Sub-theme 2.3: MHPs identities’ impact on help-seeking

While MHPs generally found it difficult to pinpoint, most agreed that markers of their personal identity—such as gender, skin colour, nationality, age, or cultural background—can influence whether staff choose to seek their support. However, many emphasized that this influence tends to be short-lived. As one MHP explained, “your gender, your skin colour, your attitude in general is a factor,” but once clients “test you, and they find that you are like a real professional person, then it does not matter” (Participant 1).

Gender was repeatedly highlighted by both male and female MHPs as a particularly significant factor, shaping both organisational expectations and client preferences. One MHP noted that their organisation often preferred women for counselling roles, as the profession is commonly associated with “providing care support (…) like a mother type of” role, embodying “feminine traits so to speak” (Participant 3). Another reflected on how gender-based discrimination in the humanitarian sector — mainly targeted at women — may shape who feels comfortable opening up: “Maybe that hinders their capacity to speak to [a man], because they would prefer to speak to somebody from their gender, who can understand them and not being a man” (Participant 7). In some contexts, cultural norms around privacy further reinforced this dynamic: “You do not talk to a man about some private stuff in some culture if you are a woman, or vice versa” (Participant 8).

Intersections of markers of identity also shaped how MHPs were perceived and how easily trust was established. Several noted that being “older” (Participant 5), “seniority” (Participant 4), “European” (Participant 5, Participant 7), “American” (Participant 7), or “white” (Participant 3, Participant 4, Participant 7) often confers an assumption of competence and authority, reducing the pressure to prove oneself. Conversely, one MHP described how ambiguity around their background allowed them to “blend in,” avoiding fixed assumptions (Participant 7). Others observed that staff might feel more understood by MHPs perceived to share similar lived experiences. However, cultural perceptions about counselling itself can also affect help-seeking, with one MHP noting that in some regions “people do not believe in counselling,” (Participant 9) which can delay engagement regardless of the counsellor’s identity.

#### 3.2.4 Sub-theme 2.4: MHPs’ personal experiences of racism

Closely related to the previous sub-theme are findings on MHPs’ own experiences of racism. Their reflections varied widely — ranging from negation or uncertainty, to discomfort and subtle interpersonal moments. One MHP, for example, said: “I do not know. I am not sure. I do not remember things.” With regard to their own sensitivity to the issue, they added: “I’m hypersensitive to many matters, including racism, (…) but never had this coming to me” (Participant 7).

Another MHP offered a more nuanced account, questioning how to define racism in moments of cultural stereotyping. Recalling an incident where colleagues joked that they “really dance like a white,” they reflected: “Is this racism or not? (…) I’m joking, we are joking (…). But because you are implying I’m not dancing well, you know?” Rather than framing such comments strictly as racist, they interpreted them through the lens of mutual recognition: “Sometimes it’s not racism—it’s just a matter of identity, to acknowledge (…) where I’m from or where you are from” (Participant 8). Adding a more subtle, emotional layer to these dynamics, one MHP described being perceived with hesitancy, or only when clients have reached rock bottom, despite being recognized as capable:

*“I notice that people come to me because they know I can do things—but it’s almost like, ‘I have no other option.’ There’s a certain hesitancy. It’s not obvious or spoken, but I sense it. I do not have anything concrete—it’s more a feeling, that unspoken behaviour, like they come as a last resort”* (Participant 3).

Illustrating the specifics of the humanitarian sector, one MHP emphasized that they had never thought much about race prior to working in that environment. Coming from a country with a wide range of skin tones, they explained: “I never ever had this concept of this person (…). I do not see the skin colour of the people. I’m very colorblind about this issue naturally.” However, upon entering the organisation, they encountered unexpected racialized dynamics: “The first day I joined (…), I started to hear these things (…) ‘Your boss is a West African, so be careful’” (Participant 1).

This marked the first time that racial labels and warnings had entered their professional awareness.

### 3.3 Theme 3: organisational responses and constraints

#### 3.3.1 Sub-theme 3.1: evaluation of organisational responses to racism and mental health

MHPs confirmed a growing institutional awareness of racism, particularly since 2020 and the racial justice uprisings in the US and beyond. One noted that “speaking about this in a structural way has been a progress over the past 2 years,” although this was framed as largely reactive—“we were reacting to the George Floyd phenomenon” (Participant 7).

There was broad agreement that existing organisational initiatives—such as policies, trainings, webinars, and emails—tend to focus on broader DEI efforts rather than specifically addressing racism. These efforts were described as “very theoretical,” which one MHP found “beautiful to hear” but ultimately disconnected from the lived experiences of staff: “the reality is completely different, just like a day and night” (Participant 3). Moreover, MHPs pointed out that even when racism is addressed, it is not linked to organisational discourse on staff health and well-being. The psychological impacts are “completely forgotten” in institutional responses (Participant 3). One MHP acknowledged that the organisation “wants an inclusive environment for everyone with no discrimination” and highlighted “the policies they are putting in place, for sure, give to some minority group a feeling, a perception to be more accepted, or to have the doors to knock in case of problem” (Participant 8). Others, however, rather emphasised the critical gap created by the absence of sustained, concrete action —particularly in connecting racism to mental health.

The format and delivery of anti-racism trainings raised additional concerns. Online formats, in particular, were seen as encouraging disengagement, as staff can complete them passively. This renders them more of a “ticking the box” exercise than a meaningful learning opportunity (Participant 3). At the same time, this MHP recalled that in a rare open, face to face discussion on racism, reactions varied: some national staff responded, “We do not understand why you are saying we are being discriminated against,” highlighting how experiences and perceptions of racism differ by country and context (Participant 3).

Another critique concerned the perceived disconnect between organisational DEI approaches and the “multicultural aspects” of the HD sector. One MHP argued that these approaches are shaped by external donor pressures and Western frameworks: “It speaks well to the (…) donors’ requirements, to the white mentality. It does not speak to what are the real problems” (Participant 7).

MHPs also pointed to persistent challenges with organisational reporting mechanisms. Although such systems were in place, many questioned their credibility and impact. One MHP explained that staff “are scared normally of retaliation. (…) Their biggest fear is that they try to do something, then there are no consequences for the person who is harming them.” The same participant added that staff fear being “labelled as the troublemaker,” “crazy” or “too sensitive” (Participant 5), even when formal apologies are issued. Furthermore, one MHP voiced their own frustration with the institutional response system: “I get frustrated sometimes, because (…) our response organisational system is not that fast in terms of how to investigate, and so on” (Participant 7). In reflecting on this situation, they positioned it as outside their control, distancing themselves from personal responsibility: “But the reality is, it’s not something related to my capacity or to my role” (Participant 7).

#### 3.3.2 Sub-theme 3.2: what organisations should do

To address the lived realities of staff and raise awareness about the link between racism and mental health, MHPs called for more grounded, context-sensitive action across multiple dimensions—interventions that would move beyond symbolic gestures to create tangible impact. These included “face to face (…) awareness discussions,” particularly on the human values people carry (Participant 3), and fostering “a more grounded engagement with staff” (Participant 7). Others proposed establishing “common spaces to talk” (Participant 10) and ensuring “we have (…) policies and we have (…) consequences” for violations (Participant 5).

There was a strong emphasis on the importance of cultural awareness and tailored training. One MHP suggested allocating more time to cultural briefings, arguing that acknowledging differences and learning about others’ cultures was among the most effective approaches. Training managers, in particular, was deemed essential: “Managers are not aware of simple examples that are hidden under the surface” (Participant 4). Another MHP noted that some managers acted in discriminatory ways “without understanding,” assuming “I’m not racist, I really did not mean it,” while still exercising “absolute power” (Participant 1). Similarly, a participant stressed the need to “build more culturally informed management styles,” arguing that staff were managed in vastly different cultural contexts—“in Afghanistan like [in] Colombia”—without recognising how norms around “professionalism” may vary (Participant 7).

Suggestions around reporting systems included simplifying procedures and ensuring a timely, meaningful response. One MHP proposed “making more easy mechanisms to report and to address this,” noting that “it’s not about reporting, it’s about being able to respond in a fast and a prompt way” (Participant 7). Others expressed doubt about the effectiveness of formal structures, citing fear of retaliation and lack of consequences. As a more approachable alternative, one MHP advocated for informal conversations—“a cup of coffee conversation” (Participant 4)—as a way to express concerns early in a non-punitive, empathetic setting before escalating issues formally.

The need to shift from reactive to proactive and integrated organisational cultures was another recurrent theme. One MHP called for those working in staff mental health to be fully recognised and included in broader institutional reflections: “We are not here only to react (…) but [should be] part of the reflection at the bottom of an organisational culture” (Participant 8). Others underlined the importance of systemic transformation, including within hiring practices. As one MHP explained:

*‘We can change (…) the way we recruit people. People from the field they (…) want to come for HQ (…). I think that the field is diverse, but I think in HQ, we should have a workforce that is more diverse. And then people thinking differently, we also have more creativity and more space for debate. And not only, you know, a lot of people thinking the same way’ (Participant 5).*

Finally, it was suggested that even MHPs themselves may require support in developing culturally competent practice. One MHP emphasised the need to “train counsellors on transcultural issues,” noting that differences in educational backgrounds and training meant not all professionals had the same skills: “So we should standardise it among all the counsellors” (Participant 1).

## 4 Discussion

The aim of this study was to explore in-house MHPs’ perspectives on racism within HD organisations, including its impacts on staff mental health, their professional role in addressing these effects, and their evaluation of organisational responses and needed change. Consistent with qualitative scholarship on racism that treats lived experience as a valid source of knowledge (Fuchs et al., 2025; Piwoni, 2025), and with research recognising the subjective experience of racism as a psychosocial stressor relevant to mental health (Pascoe and Smart Richman, 2009), this study focused on how MHPs themselves understand and interpret racism and its consequences, rather than assessing accounts against legal definitions of discrimination. At the same time, it is important to note that some accounts may also reflect what, in more formal definitions, would be considered related forms of discrimination (e.g., xenophobia), highlighting the complexity and at times blurred boundaries between these phenomena in practice. Relatedly, certain statements illustrate that race is not merely constructed through bodily traits, but is also deeply spatialized and tied to geographical imaginaries, where nationality or region of origin (e.g., “West African”) becomes a proxy for racialised expectations. This reflects a broader recognition in critical race scholarship that racialisation operates through spatial as well as bodily forms of differentiation (e.g., Ferreira Da Silva, 2007).

The qualitative analysis of semi-structured interviews with MHPs based across different organisations and multiple regions identified three main themes aligned with the study’s aims and research questions. MHPs reported that discussions about racism have become more structurally framed and that initiatives such as trainings and policies have proliferated within their organisations. Nevertheless, as expected, racism continued to be described as a persistent yet insufficiently addressed feature of organisational life, mirroring recent studies on racism in HD organisations (e.g., Aid agency actions, 2021; Bian, 2022; Slim, 2022; Prom-Jackson, 2023; Strohmeier et al., 2024b). MHPs described a range of structural manifestations of racism, including the longstanding divide between national and international staff and its implications for career progression, among other inequities. While this structural divide has long been widely discussed in literature on inequality and reform in the HD sector (e.g., Houldey, 2017; Holden et al., 2018; Strohmeier et al., 2019; Bian, 2022; James, 2022; Slim, 2022; Khan et al., 2023; Khera et al., 2024), participants also pointed to the influential role of donors: staff from main UN donor countries were described as receiving greater institutional protection and support during crises, and recruitment practices were seen as favouring nationals of major funding states. Furthermore, DEI initiatives were viewed as matching “Western frameworks” and the “white mentality,” thereby risking the reproduction of Eurocentric norms even as they are framed as inclusive. This highlights, once more, how “donor power” (Strohmeier et al., 2024a) not only shapes funding flows and sectorial priorities, but also appears to permeate organisational practices and staff experiences within the HD sector (Fechter and Hindman, 2011; Allen, 2015; Slim, 2022; Strohmeier et al., 2024a).

Also unsurprisingly, MHPs’ accounts indicate that racism can have profound effects on staff, describing a range of psychological difficulties. This is consistent with a substantial body of research linking racism to adverse mental health outcomes across multiple population groups (e.g., Bird et al., 2021; Schouler-Ocak and Moran, 2022; Webb et al., 2022). Importantly, MHPs highlighted the cumulative burden of everyday exposure to racism. Indeed, harm does not require overt or extreme incidents, as repeated experiences of microaggressions and subtle discrimination can, over time, lead to significant psychological distress (Pierce, 1970; Vines et al., 2017; Barber et al., 2019; Schouler-Ocak et al., 2021). Coping and help-seeking behaviours of staff, according to MHPs, are shaped by a range of individual and organisational factors, including personal cultural and religious backgrounds, sector-specific beliefs about strength—described in the literature as “cowboy culture” (Weymann, 2020) or a “macho/martyr” attitude (Cockcroft-McKay and Eiroa-Orosa, 2021)—as well as the availability and perceived safety of formal support options. Notably, MHPs observed that few staff approach them explicitly to seek support for racism-related concerns. This mirrors general findings from research with UN staff that shows that many of those experiencing or witnessing discrimination take no formal action due to fear and lack of trust in the organisation (Task Force on Addressing Racism and Promoting Dignity for All in the United Nations Secretariat, 2021; Strohmeier et al., 2024b). One implication of this privatisation of coping is that it contributes to obscuring the scale of the problem, thereby unintentionally sustaining a dynamic in which individuals are left to manage the effects of collective burdens largely on their own.

With regard to their own identity, MHPs reported that factors such as gender, skin colour, nationality, age, cultural background, and other relevant social identities influenced interactions with clients initially, but typically receded in importance once professional competence and trust were established. While not surprising, these dynamics underscore the importance of cultural awareness and competence among MHPs in HD contexts, where workforce diversity and related power asymmetries make it essential for practitioners to reflect on their positionality and own (unconscious) biases (Bintley and George, 2023). Furthermore, the findings suggest that—although MHPs did not articulate this explicitly—they occupy a complex professional position: they are tasked with responding to individual psychological distress while recognising that such distress is shaped by broader structural and interpersonal forms of racism that exceed the scope of individual therapy and remain insufficiently addressed by the very organisations that employ them. Sustained exposure to such tensions can lead to moral distress and injury (Khera et al., 2024), compassion fatigue (Stamm, 2005), and secondary traumatic stress and job burnout (Cieslak et al., 2014), all of which can affect practitioners’ mental health and, in turn, the quality of care they provide (Noor et al., 2025). Although none of these effects were reported by participants or observed in the interviews, recognising this potential risk is important.

In light of this complex position, supporting in-house MHPs is essential. Although the interviews were exploratory and not designed as awareness-raising exercises, several participants described them as valuable opportunities for reflection and professional learning, suggesting that structured spaces for dialogue—where not yet available—may further enrich existing practice. At the same time, the MHPs interviewed in this study demonstrated awareness of the relations between racism and mental health, informed by professional training and/or personal experience. Yet, offering regular supervision, intervision, and continuing professional development opportunities that incorporate anti-racist perspectives, joint reflection on (unconscious) biases, and attention to intersectionality may support MHPs in their ongoing efforts to provide robust, contextually attuned support to staff. Additionally, complementary resources, such as the ETHICA-4P Clinical Practice Toolkit (Calia et al., 2025), can further assist MHPs in delivering culturally adapted interventions by providing practical frameworks for tailoring mental health support to clients’ social, cultural, and ethical contexts, thereby enhancing the relevance and responsiveness of care (Rathod et al., 2018).

Wang et al. (2024), drawing on observations beyond the HD sector, report that organisational awareness of the connections between discrimination and (mental) health remains limited. This study’s findings echo this in an important way. While, as just noted, MHPs themselves showed awareness of these dynamics, the results indicate that organisational anti-racism and staff mental health initiatives are often developed and implemented in parallel rather than in an integrated manner. Although this is common across many organisations, and reflected in the institutional policies reviewed (Task Force on Addressing Racism and Promoting Dignity for All in the United Nations Secretariat, 2021; United Nations, 2024), such siloing in policy and practice is likely to constrain the effectiveness of both anti-racism efforts and mental-health initiatives, as integrated approaches and intersectoral collaboration are increasingly associated with more effective and sustainable organisational and health outcomes (Rudolph et al., 2013; Mental Health at Work, 2024; Wang et al., 2024). Instead, anti-racism and staff mental health initiatives—and the links between them—must be integrated and sustainably embedded in the structural and cultural fabric of HD organisations, across both policy and practice, to foster environments characterised by awareness, accountability, and psychological safety at all levels: rather than treating it as a peripheral issue, organisations must recognize anti-racism as central to their mission of promoting staff mental health and well-being. Building on existing literature and their professional experience as public health researchers and DEI consultants, Wang et al. (2024) propose five “guideposts” for integrating wellbeing and DEI efforts in organisational contexts: raising awareness of the relationship between DEI and employee health; establishing shared organisational goals that integrate DEI and wellbeing and investing resources accordingly; measuring and analysing wellbeing across demographic groups to assess equity; recognising differences in employees’ experiences and needs and tailoring support; and prioritising systemic approaches to promote employee health and organisational DEI. Implementing these guideposts, the authors argue, can lead to multiple benefits for organisations, “including efficiency, strategic alignment and consistent messaging, comprehensive employee support, enhanced employee engagement, and improved recruitment and retention” (Wang et al., 2024, p. 1093). For MHPs, this would mean not only supporting individuals affected by racism, but also being more meaningfully included in broader institutional efforts to create safer, more equitable workplaces—helping to identify and prevent structural and interpersonal conditions that give rise to harm.

As, among others, Devakumar et al. (2025) and Srivastava (2024) point out, not all, but much of the recent literature on racism and anti-racism, including in the workplace, originates from the United States as well as other Western contexts. Similarly, the bulk of the psychosocial support services HD organisations currently offer—such as one-on-one counselling—are largely shaped by Euro-American understandings of mental health and healing, potentially leaving significant segments of the workforce underserved (Strohmeier and Ganter-Argast, 2026). It may therefore be timely to consider complementing these approaches with interventions that are equity-oriented, culturally adaptable, and effective, and that have great potential to alleviate individual stress while also enhancing team cohesion. In this context, arts-based interventions (ABIs) may represent one useful avenue to support existing services: ABIs such as visual arts, dance, or music-making, facilitated by trained professionals, are increasingly recognised as valuable approaches for processing (racial) trauma, particularly in cross-cultural or multilingual settings where verbal communication may be limited (Fancourt and Finn, 2019; Guerra et al., 2024; Strohmeier and Ganter-Argast, 2026). Applicable in both individual and group settings, these practices can foster resilience while also creating spaces in which silence may be broken and shared understanding can emerge. In this sense, mental health support extends beyond individual healing; it can serve as a conduit for transforming private suffering into collective awareness and action. As presented in a different article, the MHPs interviewed for this study showed strong initial support for ABIs as complementary approaches to existing staff-care systems (Strohmeier and Ganter-Argast, 2026). Importantly, however, ABIs—like any other staff-care intervention — should not replace structural reform, but rather support it by helping connect individual and group experiences with processes of institutional change.

This study has limitations. First, the sample size is relatively small and the sampling strategy—although widely used in qualitative research (Ting et al., 2025) – is prone to bias. The sample was also gender-imbalanced, which may have shaped the perspectives represented in the data. Furthermore, the data rely on self-reported experiences, which are inherently subjective and may be influenced by limitations in recall. However, the data collected are rich, MHPs constitute a hard-to-reach population, and to our knowledge this is the first study to examine racism and mental health within HD organisations from the perspective of this specific occupation group. Second, detailed demographic and positional information is relevant in research on racism, as it provides important contextual grounding. Yet, given the small and specialised sample, reporting such details would have substantially increased the risk of deductive disclosure, thereby compromising participant confidentiality and trust. We therefore chose to prioritise participant protection over detailed sample description. This decision aligns with established guidance on managing the risk of deductive disclosure in qualitative research (Ethical considerations associated, 2022).

In conclusion, this study makes three main contributions. First, it documents how racism is experienced and understood in HD organisations from the vantage point of in-house MHPs. Second, it shows how racism-related distress is both widespread and largely privatised, with limited use of formal in-house psychosocial support, thereby obscuring the true scale of the problem and shifting responsibility onto individuals. Third, it demonstrates that organisational responses to racism and staff wellbeing remain institutionally siloed, with anti-racism and mental-health initiatives typically pursued in parallel rather than as an integrated strategy. Taken together, this study’s findings suggest that racism in HD organisations cannot be addressed solely through standalone DEI or anti-racism initiatives, nor through individualised staff-care programmes. Instead, racism needs to be recognised as a core occupational health and safety issue, with anti-racist organisational change and staff mental-health strategies designed and implemented in tandem. While many of this study’s findings align with broader scholarship, they have not previously been systematically examined within the HD workforce or from the perspective of in-house MHPs. This study therefore fills a critical gap by establishing an evidence base where none previously existed.

## Data Availability

The datasets presented in this article are not readily available because this manuscript is based on qualitative data. Making the whole interview transcripts publicly available would violate the confidentiality agreement with participants. Requests to access the datasets should be directed to HS, hannah.strohmeier@charite.de.
